# Predicting autosomal dominant polycystic kidney disease progression: review of promising Serum and urine biomarkers

**DOI:** 10.3389/fped.2023.1274435

**Published:** 2023-11-10

**Authors:** Iva Sorić Hosman, Andrea Cvitković Roić, Margareta Fištrek Prlić, Ivana Vuković Brinar, Lovro Lamot

**Affiliations:** ^1^ Department of Pediatrics, General Hospital Zadar, Zadar, Croatia; ^2^ Department of Nephrology and Urology, Clinic for Pediatric Medicine Helena, Zagreb, Croatia; ^3^ Department of Pediatrics, Faculty of Medicine, Josip Juraj Strossmayer University of Osijek, Osijek, Croatia; ^4^ Department of Pediatrics, Faculty of Medicine, University of Rijeka, Rijeka, Croatia; ^5^ Department of Nephrology, Hypertension, Dialysis and Transplantation, University Hospital Centre Zagreb, Zagreb, Croatia; ^6^ Department of Internal Medicine, School of Medicine, University of Zagreb, Zagreb, Croatia; ^7^ Division of Nephrology, Dialysis and Transplantation, Department of Pediatrics, University Hospital Centre Zagreb, Zagreb, Croatia; ^8^ Department of Pediatrics, School of Medicine, University of Zagreb, Zagreb, Croatia

**Keywords:** autosomal dominant polycystic kidney disease (ADPKD), prognostic biomarker, predictive biomarker, copeptin, angiotensinogen (AGT), urinary biomarkers, urinary proteomics, urinary metabolomics

## Abstract

Autosomal dominant polycystic kidney disease (ADPKD) is one of the leading causes of end-stage renal disease. In spite of the recent tremendous progress in the understanding of ADPKD pathogenesis, the molecular mechanisms of the disease remain incompletely understood. Considering emerging new targeted therapies for ADPKD, it has become crucial to disclose easily measurable and widely available biomarkers for identifying patients with future rapid disease progression. This review encompasses all the research with a shared goal of identifying promising serum or urine biomarkers for predicting ADPKD progression or response to therapy. The rate of the ADPKD progress varies significantly between patients. The phenotypic variability is only partly explained by the underlying genetic lesion diversity. Considering significant decline in kidney function in ADPKD is not usually evident until at least 50% of the parenchyma has been destroyed, conventional kidney function measures, such as glomerular filtration rate (GFR), are not suitable for monitoring disease progression in ADPKD, particularly in its early stages. Since polycystic kidney enlargement usually precedes the decline in GFR, height-adjusted total kidney volume (ht-TKV) has been accepted as an early biomarker for assessing disease severity in ADPKD patients. However, since measuring ht-TKV is time-consuming and observer-dependent, the identification of a sensitive and quickly measurable biomarker is of a great interest for everyday clinical practice. Throughout the last decade, due to development of proteomic and metabolomic techniques and the enlightenment of multiple molecular pathways involved in the ADPKD pathogenesis, a number of urine and serum protein biomarkers have been investigated in ADPKD patients, some of which seem worth of further exploring. These include copeptin, angiotensinogen, monocyte chemoattractant protein 1, kidney injury molecule-1 and urine-to-plasma urea ratio among many others. The aim of the current review is to provide an overview of all of the published evidence on potentially clinically valuable serum and urine biomarkers that could be used for predicting disease progression or response to therapy in patients with ADPKD. Hopefully, this review will encourage future longitudinal prospective clinical studies evaluating proposed biomarkers as prognostic tools to improve management and outcome of ADPKD patients in everyday clinical practice.

## Introduction

Autosomal dominant polycystic kidney disease (ADPKD) is one of the leading causes of end-stage renal disease (ESRD) worldwide (1). This monogenic disorder, with an incidence of 1 in 400 to 1,000 live births, is characterized by gradual bilateral enlargement of kidneys caused by the sustained expansion of numerous fluid-filled epithelial cysts leading to a progressive loss of functional nephrons (2, 3). ADPKD is most commonly caused by mutations in PKD1 and PKD2 gene, accounting for approximately 78% and 15% of cases, respectively (4, 5). These genes encode proteins in primary cilia of renal tubular epithelial cells (polycystin-1 and -2). Their mutations result in defective cell proliferation, tubulogenesis and excessive fluid secretion, ultimately resulting in slowly expanding renal cysts (4). Rarely, mutations in other genes, including GANAB and DNAJB11, are found in ADPKD patients (5–7). Although affected gene and type of mutation alter the ADPKD clinical phenotype (PKD1 patients have a more severe renal impairment and faster disease progression than those with PKD2 mutations; PKD1 truncating mutations result in a more severe phenotype than PKD1 non-truncating mutations), major phenotypic differences among ADPKD patients can only partly be explained by the underlying gene mutation (8–10).

In addition to truncated PKD1 mutations, several biological and clinical characteristics of ADPKD patients have been recognized as prognostic indicators. These include gender and age at diagnosis with male gender and earlier age of disease onset being risk factors for a more rapid disease progression (11, 12). Furthermore, hypertension—which occurs in up to 80% of ADPKD patients before a significant renal function decline, hematuria, proteinuria, higher baseline kidney volume and lower baseline GFR are already established risk factors for a faster disease progression in ADPKD patients (11–14). More recently, higher baseline body mass index (>25 kg/m^2^) and metabolic syndrome have been recognized as additional modifiable risk factors for a rapid ADPKD progression (15, 16). It still remains to be elucidated whether these prognostic indicators are causal or consequential to ADPKD.

For decades, ADPKD was considered primarily adult disease. With recent improvements in ultrasound technology, it has become clear that cyst development begins in childhood or even prenatally resulting in an increased number of prenatal and infant diagnoses of ADPKD (17, 18). Even though children with ADPKD are typically asymptomatic, longitudinal magnetic resonance imaging (MRI) studies in pediatric ADPKD population have demonstrated considerable progressive kidney enlargement (19, 20). Since the rate of clinical deterioration and the age of ESRD onset in ADPKD are still unpredictable, establishing risk factors for a rapid disease progression early in childhood might allow identifying high–risk ADPKD patients in a need for an adequate early intervention.

After ESRD onset, disease progression is relatively constant and may be monitored by glomerular filtration rate (GFR) (21). However, in early disease stages, as a result of glomerular hyperfiltration, kidney function remains preserved (22, 23). In fact, renal volume increases years before GFR starts to decrease, making GFR an insensitive early marker of ADPKD progression (23, 24). Series of studies by the Consortium for Radiologic Imaging Studies of Polycystic Kidney Disease (CRISP) established height adjusted total kidney volume (htTKV), measured by MRI, as a more reliable biomarker of renal disease progression in ADPKD patients, particularly in its early stages, before extensive renal parenchymal damage has occurred (24, 25). The best predictor of disease progression currently is a prognostic model based on age-adjusted htTKV, the Mayo Imaging Classiffication (MIC), which categorizes ADPKD patients into classes 1A through 1E withhigher classes at a substantially greater risk of progression to ESRD (26). Regrettably, its prognostic value is limited in atypical cases (patients with markedly asymmetric kidneys) as well as in patients with coexisting ischemic disease. In addition, ht-TKV explains nearly 42% of the difference in GFR, indicating that other noncystic mechanisms, which are not considered in MIC, affect kidney function decline (27). More recently, ADPKD Outcomes Model (ADPKD-OM) based on disease progression equations for htTKV and estimated GFR (eGFR) has been proposed (28). Another widely used prognostic model for predicting progression to ESRD in patients with ADPKD is the Predicting Renal Outcome in Polycystic Kidney Disease (PROPKD) score which combines underlying genetic mutation and clinical risk factors (gender, presence of hypertension and/or early urological events before the age of 35) to categorize ADPKD patients as low, intermediate or high risk of progression to ESRD (29). This prognostic model is inapplicable in patients younger than 35 years and negative for PKD1 or PKD2 mutation.

Although MIC, ADPKD-OM and PROPKD are nowadays the most commonly used prognostic models in ADPKD clinical trials, all of these predictive scores remain limited by availability, high price and expertise mandatory for obtaining such parameters and therefore are not useful in everyday clinical practice (30, 31). Furthermore, htTKV is not a reliable biomarker for assessing response to certain therapeutic agents in PKD patients since a number of studies demonstrated that reduction in the rate of TKV growth induced by these agents (mTOR inhibitors and somatostatine analogues) is not accompanied by a slower disease progression measured by eGFR (32–37). Currently, the only Food and Drug Administration-approved ADPKD therapy is tolvaptan—the vasopressin V2 receptor antagonist. Arginine vasopressin (AVP), antidiuretic hormone, plays an important role in the PKD pathogenesis as it promotes both cystic cell proliferation as well as luminal fluid secretion (38). Tolvaptan has been demonstrated to inhibit both of these effects and, consequently, slow both renal function decline and TKV growth (39). Since tolvaptan has considerable side effects including polyuria and hepatotoxicity, and is more effective when introduced in the earlier phase of ADPKD (40–42), identifying patients with a higher risk of an accelerated disease progression in an early disease stage is of the uppermost importance for selecting patients who will benefit from an early introduction of therapy. In addition to that, with emerging evidence of cellular signaling pathways which are dysregulated in ADPKD (43), there is a growing list of experimental targeted therapies under different stages of investigation (44), highlighting the need for reliable prognostic (identifying the probability of a rapid clinical progression) and predictive (identifying patients who are more likely to respond to a treatment) biomarkers in ADPKD.

For these reasons, growing number of studies are investigating multiple serum and urinary molecules, particularly those involved in underlying pathophysiology, as alternative biomarkers for predicting fast disease progression in ADPKD patients. Herein, we highlight emerging urine and serum biomarkers that have been associated with a higher risk of rapid ADPKD progression.

## Search methods

**Figure 1:**
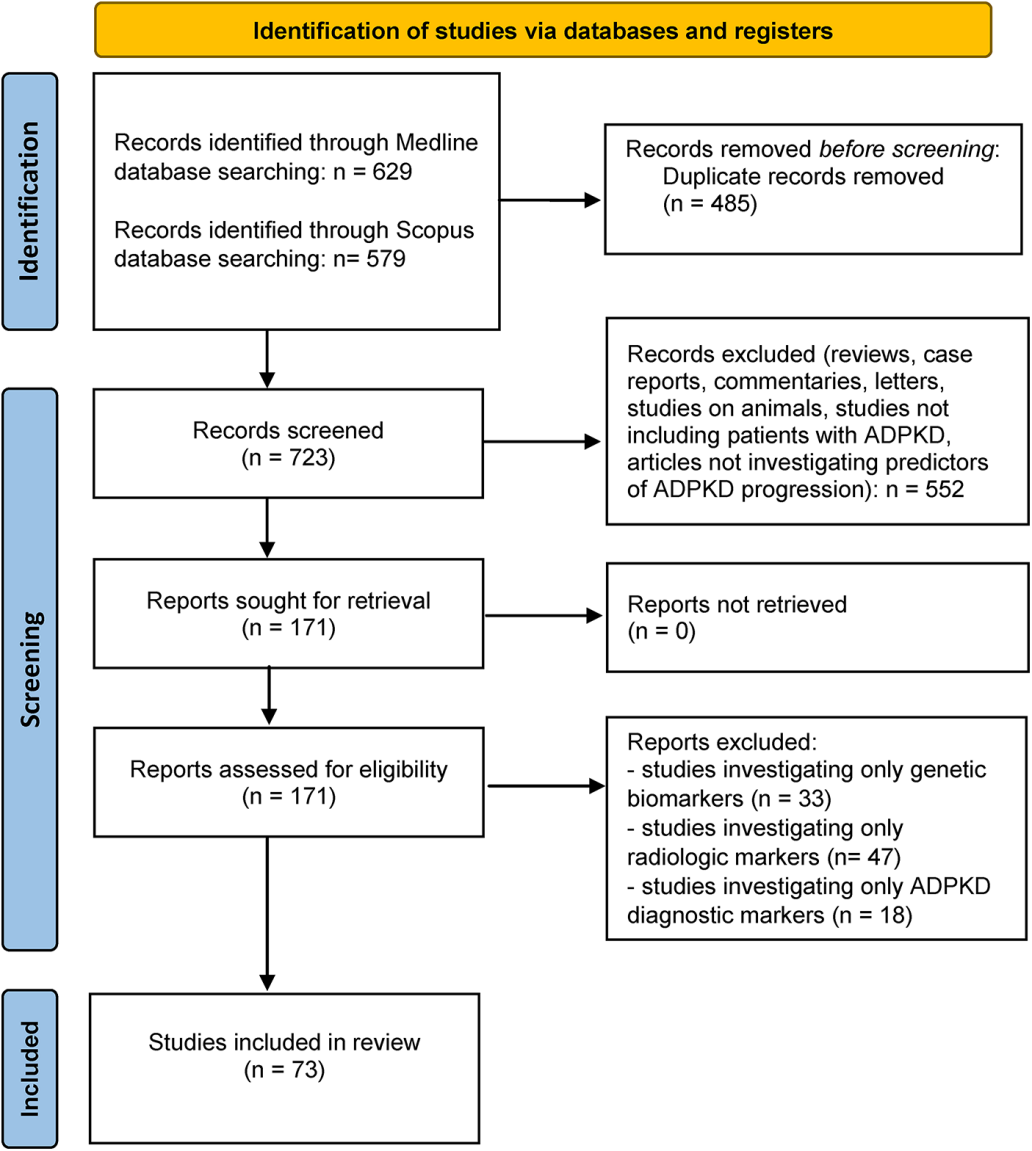
PRISMA-flowchart (40) illustrating the literature search and study selection process.

A comprehensive literature review was conducted using Medline and Scopus databases to discern published articles investigating the potential serum or urine biomarkers for predicting ADPKD progression, in accordance with the published guidelines for writing systematic reviews (45). We used the combination of search terms “polycystic kidney disease” and “marker” or “biomarker” in all fields including keywords, MeSH terms or any text word. Only available full-text articles in English published until May 1st 2023 were included in this review. Case reports, reviews, commentaries, animal studies and studies not concerning patients with PKD or not discussing potential predictors of disease progression were all excluded. The database search identified 1,208 articles of which 723 remained after the duplicate removal and 171 after title/abstract screening. In the end, after evaluating the full-text articles for eligibility, 73 full-text articles were incorporated in the present review and thoroughly analyzed. More details of the study selection process are presented in Figure 1– PRISMA flowchart. All published studies in which urine or serum biomarkers were evaluated as predictors of a rapid ADPKD progression are included in this review and summarized in Table 1.

**Table 1:** Summary of the proposed biomarkers for predicting ADPKD progression.

Proposed biomarker	Potential applicability in ADPKD patients	Limitations	Relevant studies
Copeptin	Biomarker of disease severity Biomarker of cardiovascular complications development Prognostic biomarker for rapid disease progression Predictive biomarker of response to tolvaptan therapy	Multiple lifestyle factors influence copeptin serum level; Gaps in knowledge about its pharmacokinetics and degradation; Considerable overlap in the rate of disease progression between groups with high vs. low copeptin levels	(46–55)
Apelin	Biomarker of disease severity Prognostic biomarker for rapid disease progression	Scarce evidence (only two published small cohort studies); Gaps in knowledge about its pharmacokinetics	(55, 56)
Urine to plasma urea ratio	Biomarker of disease severity Prognostic biomarker for rapid disease progression	Limited data derived from small cohort studies	(50, 57–59)
Urinary osmolality	Biomarker of disease severity Prognostic biomarker for rapid disease progression Predictive biomarker of response to therapy	Depends on water intake and the daily amount of osmoles excreted; Some studies (60) reported no predictive value	(61–63)
Urinary angiotensinogen (uAGT)	Biomarker of disease severity Biomarker of hypertension development Prognostic biomarker for rapid disease progression	Unknown whether it is a damage marker or contributor to kidney damage; Strength of the correlations observed in most studies is modest	(64–71)
Fibroblast growth factor 23 (FGF23)	Biomarker of disease severity Prognostic biomarker for rapid disease progression	Mechanism and role of FGF23 elevation in ADPKD are unknown; Some studies (69) did not show additional prognostic value of FGF23 over ht-TKV	(72–75)
Soluble urokinase plasminogen activator receptor (suPAR)	Prognostic biomarker for rapid disease progression	Scarce evidence (only one published study)	(76)
Serum bicarbonate	Prognostic biomarker for rapid disease progression	Scarce evidence (only one published study); Dependent on urinary acidification capacity (urinary ammonium excretion)	(77)
Secreted frizzled-related protein 4 (sFRP4)	Biomarker of disease severity Prognostic biomarker for rapid disease progression	Scarce evidence (only one published study)	(78)
Uric acid	Biomarker of disease severity Prognostic biomarker for rapid disease progression	Scarce evidence (only two published studies)	(79, 80)
Urinary biomarkers: *Β*-2-microglobulin (β-2MG), monocyte chemoattractant protein-1 (MCP-1), vascular endothelial growth factor (VEGF), kidney injury molecule-1 (KIM-1)	Biomarker of disease severity Prognostic biomarker for rapid disease progression	Relatively low predictive value for future ADPKD progression for each biomarker separate; Lack of measuring methods standardization; Frozen storage might decrease the measured concentration of urinary biomarkers	(81–89)
Urinary neutrophil gelatinase-associated lipocalin (NGAL)	Biomarker of disease severity Prognostic biomarker for rapid disease progression	Opposite results in terms of predictive value for future ADPKD progression; Frozen storage might decrease the measured concentration	(90–92)
Urinary CD4+ T cell count, CD206 + macrophages count and CD14 levels	Biomarkers of disease severity Prognostic biomarkesr for rapid disease progression	Scarce data (only one published small cohort study for each biomarker)	(93–95)
Asymptomatic pyuria	Prognostic biomarker for rapid disease progression	Scarce data (only one published cohort study); Infection, tumor, previous catheterization and contamination must be excluded	(96)
Urinary fetuin A	Prognostic biomarker for rapid disease progression	Scarce data (only one published small cohort study	(97)
Urinary osteopontin	Prognostic biomarker for rapid disease progression	Scarce data (only one published small cohort study	(98)
Telomere length and TERRA	Prognostic biomarker for rapid disease progression	Scarce data (only one published small cohort study); Ageing as well as physiological and psychological stressors lead to telomere shortening and increased TERRA	(99)
Metabolites identified by targeted urinary or serum metabolomics	Biomarkers of disease severity Prognostic biomarkers for rapid disease progression	Scarce data; Different metabolites are identified by different studies	(100–106)
Peptides identified by urinary proteomics	Biomarkers of disease severity Prognostic biomarkers for rapid disease progression	Marked individual variations in the urine proteome based on multiple factors; Optimization of methods for isolating/purifying EVs is required	(107–111)
MicroRNAs	Biomarkers of disease severity Prognostic biomarkers for rapid disease progression	Optimization of methods for isolating/purifying microRNAs is required	(112–114)

## Results

### Vasopressin, copeptin and apelin

One of the first clinical features in PKD patients that occurs prior to a kidney function decline is a decreased urine concentrating capacity (57, 115). Torres et al. observed that up to 60% of children with ADPKD do not condensate urine after receiving desmopressin (116). To maintain the fluid balance, concentration of AVP increases. AVP's detrimental role in the PKD pathophysiology has been well established in stimulating intracellular cAMP, which promotes cell proliferation and cyst growth [reviewed in (117)]. Furthermore, recent clinical trials have shown that suppression of AVP secretion or pharmacological AVP inhibition reduces the rate of ADPKD progression (39, 118–120). Taken all together, AVP seems to be a promising potential biomarker in ADPKD with an intriguing possibility to distinguish patients who are most likely to benefit from therapeutic intervention interfering with the AVP signaling pathway. However, over 90% of AVP in circulation is firmly bound to platelets, so its assessment might be altered by the number of platelets, their incomplete removal or pre-analytical processing steps (120). In addition, the laboratory measurement of AVP concentration is time-consuming and impractical because it is promptly removed from the circulation with a half-life of less than 30 min and is unstable in serum even when stored at −20°C (121). On the other hand, copeptin, the glycosylated C-terminal portion of the AVP precursor peptide, which is produced and released into the circulation in equimolar amounts as AVP, is stable for up to 14 days in serum at a room temperature and can be quickly measured (0.5–2.5 h) (122, 123). Studies have shown that copeptin reflects AVP serum concentration under different physiologic and pathophysiologic circumstances (122, 124–126), making it a reliable surrogate biomarker of AVP secretion in clinical practice.

In ADPKD, copeptin levels are elevated and strongly correlate with serum creatinine (60), but are comparable to copeptin levels in patients with other types of CKD (127). Hence, baseline copeptin level is not diagnostic of ADPKD. However, serum copeptin level seems to have a role as a biomarker of ADPKD severity and prognosis. Boertin et al. (46) reported a significant association of copeptin levels with ADPKD severity in a prospective study of 79 ADPKD patients (baseline GFR 96.8 ± 18.2 ml/min/1.73 m^2^) followed-up for 11 years. Serum copeptin level was inversely correlated with the renal function decline, even after adjusting for patients' age, gender, blood pressure and baseline eGFR values. Patients with a higher baseline copeptin (above the median of 2.7 pmol/L) started renal replacement therapy during follow-up. These findings confirmed the results of a previous cross-sectional study of 102 ADPKD patients in which copeptin was found to be associated positively with TKV and albuminuria and negatively with GFR and effective renal blood flow (47). Since results of these two studies (46, 47) were limited either by a small sample size or the fact that only GFR and not TKV was evaluated as a kidney outcome measure, the authors validated the reported associations in a longitudinal, observational study of a large, well phenotyped cohort of 241 early stage ADPKD patients followed-up for 8.5 years (48). This study included TKV measured by MRI for assessing disease progression in each patient. Data from this study showed that higher baseline copeptin levels are significantly associated with a more rapid disease progression expressed as change in both TKV and GFR over time, independently of patient's age, gender or kidney risk factors. However, it must be noted that hydration status at time of copeptin assessment was not standardized, what might have influenced results. The same author group validated the aforementioned results in another longitudinal study including 55 ADPKD patients with 2.8 years long follow-up (49). Contrary to plasma and serum osmolality, baseline serum copeptin concentration was significantly associated with future change in eGFR and, therefore, superior to both plasma and serum osmolality in predicting disease progression.

Plasma osmolality is the most significant physiological stimulant of AVP and copeptin secretion and studies in non-ADPKD patients have shown that plasma osmolality levels are directly proportional to those of copeptin (128, 129). Surprisingly, in most studies on ADPKD patients, no correlation between serum copeptin and plasma osmolality was found (48, 49). These data suggest that, in ADPKD, release of copeptin is not under physiological control by plasma osmolality. However, one earlier study (46) reported an association between copeptin levels and plasma osmolality in ADPKD patients, but patients had a broader GFR range (different disease stages). These contradictory results make it challenging to elucidate whether the physiological connection between plasma osmolality and copeptin is disrupted in ADPKD or not. The authors suggested that, in patients with lower GFR, the medullary urea gradient is defective because of a deficit of functioning nephrons, interstitial fibrosis and destruction of the medullary tissue caused by cysts. These suggestions have been supported by a report showing that patients with lower GFR have decreased urinary osmolarity and increased urinary volume compared to patients with normal renal function (130) and a report of an association between defective renal concentrating capacity and the severity of the renal parenchyma destruction caused by cysts (115). Consequently, higher AVP levels are necessary to maintain fluid balance and these higher AVP levels stimulate further disease progression closing a vicious circle in ADPKD. This hypothesis may provide a feasible explanation for the ADPKD progression in patients in whom GFR remains stable for a long time despite the development of cysts, but then promptly enters a phase with a rapid GFR decline.

To investigate whether increased copeptin serum levels emerge from a reduced kidney clearance or as a compensation for diminished concentrating capacity, Zittema et al. (50) compared copeptin levels between 122 ADPKD patients and 134 healthy kidney donors (in whom total GFR decreases despite having a functionally normal kidney) before and after donation. Median copeptin was initially significantly higher in patients than in donors (both pre- and post-donation). Furthermore, copeptin levels correlated with eGFR only in ADPKD patients (19% increase in copeptin per 10 ml/min/1.73 m^2^ decrease in GFR). Surprisingly, copeptin levels in donors did not change after donation, regardless of a significant GFR decline. Based on these findings the authors suggested that, in ADPKD, kidney damage and reduced urine concentrating capacity determine copeptin levels and that kidney function *per se* is not the leading determinant of the serum copeptin concentration. Therefore, changes in copeptin levels precede decline in GFR. These observations are limited by a lack of fluid and dietary intake standardization at the time of serum sample collection, as well as a lack of a control group with patients with a non-ADPKD CKD.

All of the aforementioned studies interrogated serum copeptin levels in adult ADPKD patients. We found only one study investigating serum copeptin in pediatric ADPKD population in which no significant difference in plasma copeptin between patients (*n* = 53) and healthy controls (*n* = 53) was found (51), even though lower urine and plasma osmolality was noticed in patients. The observed alterations might be due to an increased water intake before taking blood samples as advised to patients by study authors. The only one study evaluating urinary copeptin that was found included 50 ADPKD patients and reported moderate correlations between urinary copeptin and ht-TKV and eGFR (*P* = 0.008 and *P* = 0.036, respectively) (52). Therefore, the questions of utility of copeptin in pediatric ADPKD population as well as utility of urinary copeptin in general ADPKD population deserve further investigation.

Besides its correlation with disease severity and progression, higher copeptin levels have been associated with cardiovascular disease (CVD) manifestations (hypertensive vascular disease, atherosclerosis and endothelial dysfunction) in ADPKD patients. A study including 202 early stage ADPKD patients (normal eGFR) with a 3-year long follow-up, demonstrated that CVD develops even in ADPKD patients with preserved renal function and progresses faster than renal function decline (estimated by the eGFR decrease) (53). On top of that, serum copeptin levels were increased before kidney function deterioration in these patients, while the degree of increase in copeptin level correlated with CVD markers and predicted a future eGFR decline. The results of this study are limited by a lack of genetic profiling and radiologic (TKV) evaluation of patients.

Gansevoort et al. (54) investigated, in addition to validating baseline copeptin correlation with the rate of future ADPKD progression, whether baseline copeptin level and treatment-induced change in copeptin levels correlate with tolvaptan treatment efficacy in 1,280 ADPKD patients. Greater treatment effect (slower disease progression estimated by change in kidney growth rate and eGFR decline) was noted in tolvaptan-treated subjects with higher baseline copeptin levels. Tolvaptan-treated subjects with a larger percentage increase in copeptin levels during first three weeks had a more favorable disease course, with less kidney growth and eGFR decline during three year follow-up. Therefore, copeptin might be a promising biomarker for predicting outcome and tolvaptan treatment efficacy in ADPKD. Of notice, blood sampling for copeptin measurement was not done in a standardized setting (not all patients were fasting), which might have led to alterations of copeptin concentration in this study.

Lacqueniti et al. (55) corroborated copeptin as a predictive biomarker of disease progression in a cohort of 52 ADPKD patients followed up for 2 years. In addition to copeptin, the authors investigated the role of apelin as a predictor of ADPKD progression. Apelin is colocalized with AVP in magnocellular neurons (131). Water deprivation causes a substantial increase in hypothalamic apelin levels that is mirrored by plasma apelin level decline, indicating that AVP and apelin are inversely regulated (131). Indeed, both Leierer et al. (56) and Lacqueniti et al. (55) reported decreased plasma apelin concentration in ADPKD patients compared to age-, gender- and eGFR-matched healthy controls. In addition, Lacqueniti et al. (55) found that plasma apelin level is conversely correlated with plasma copeptin level and decreases gradually with the deterioration of renal function. Furthermore, apelin correlated better with both change in GFR and TKV than with urine osmolality and was found to be superior to copeptin as a predictor of disease progression. However, since these results were obtained in a single-center study including only a small cohort of 37 ADPKD patients, future studies in wider cohorts are needed to corroborate apelin as a predictive biomarker in ADPKD.

The use of copeptin as a biomarker has a few limitations. Copeptin levels are significantly higher in males compared to females, but are not strongly correlated with age (123, 132). In both healthy individuals and CKD patients, an increase in total fluid intake significantly reduces serum copeptin level (133, 134). In addition, the type of fluid consumed also influences copeptin plasma level (135). To overcome this obstacle, Mannix et al. (136) developed and validated the semi-quantitative beverage frequency questionnaire (BFQ) as a reliable tool to assess the fluid intake of the ADPKD population. Nonetheless, various other factors may alter the basal levels of copeptin including other dietary factors (sodium, potassium, protein and alcohol intake), smoking and strenuous exercise (123, 137). Additional limitations for a wide use of copeptin as a biomarker are gaps in knowledge about its pharmacokinetics and degradation, which reportedly differ from those of AVP (138). This is of a particular importance for interpreting copeptin values in the setting of impaired renal function, as GFR decline correlates better with serum copeptin than with serum AVP level (60). Additionally, it is not known whether decreased eGFR influences the level of urinary copeptin (52). The relative significance of each of these factors in altering copeptin serum levels and how to address them prior to blood sampling should be standardized for further studies. Finally, in most of the studies reviewed above there was a substantial overlap in the rate of disease progression between groups with high vs. low copeptin levels, indicating that copeptin level as a predictor of an accelerated disease progression might be reliable only in case of a low or high value, or in addition to conventional prognostic biomarkers, such as TKV and genotype.

Conclusively, analyzed studies uniformly suggest that copeptin could be a valuable early biomarker for identifying ADPKD patients with a greater risk of a rapid disease progression. Future studies on ADPKD patients with a standardized fluid and dietary intake should investigate whether assessment of serum or urine copeptin adds an extra value in predicting ADPKD progression over commonly used eGFR and TKV and whether copeptin could serve as a predictive biomarker for selecting ADPKD patients who will doubtlessly benefit from AVP-modifying therapies.

### Urine to plasma urea ratio and urine osmolality

Impaired urine concentrating capacity can be observed in patients with ADPKD even before kidney function starts to decline (57, 115). Unfortunately, to determine the urine-concentrating capacity, a prolonged water deprivation test needs to be performed, which makes it impossible to incorporate this analysis in everyday clinical practice. Bankir and Bichet (58) suggested that the defective concentrating capacity is a consequence of an urea-selective concentrating impairment caused by cystic destruction of the medullary architecture, and they proposed urine to plasma urea ratio (U/P urea) as a measure of a reduced concentrating capacity in ADPKD. Using data from water deprivation tests in patients with different stages of ADPKD, two subsequent studies (57, 59) confirmed that baseline U/P urea significantly correlates with maximal urine osmolality during water deprivation.

Furthermore, Zittema et al. (50) found an inverse correlation between U/P urea and copeptin levels in patients with APDKD. This finding was in contrast to the positive association found in donors. Surprisingly, in a multivariable regression analysis adjusted for age and sex, U/P urea was associated with TKV while GFR was not, suggesting that the urine concentrating capacity estimated by U/P urea may be an earlier and a more sensitive marker of ADPKD severity than GFR. More recently, Heida et al. (59) reported that U/P urea is significantly associated with the rate of eGFR decline in a study conducted on 583 ADPKD patients with a median follow-up of 4 years. Namely, for every 10 units decrease in U/P urea ratio the odds ratio of rapidly progressive disease was 1.35. Impressively, a combined risk score of U/P urea, Mayo classification and PKD mutation predicted accelerated disease progression better than each of the predictors individually.

Low urinary osmolality (uOsm), another surrogate marker of the impaired maximal urine-concentrating capacity, emerged as an independent risk factor for a rapid GFR decline in a retrospective analysis of 139 ADPKD patients (61). The predictive role of baseline 24-h uOsm for faster ADPKD progression (measured as both decline in GFR and increase in TKV) was validated in an extended study of the original CRISP cohort (*n* = 203, 6 years follow-up) (62). Moreover, uOsm seems to have a role as a predictive biomarker of response to tolvaptan in ADPKD patients. Devuyst et al. (63) assessed uOsm at baseline and after long-term administration of tolvaptan in a large cohort of ADPKD patients with preserved renal function. Subjects with a greater suppression in uOsm had two times less clinical progression events compared to those with the less pronounced uOsm suppression. Therefore, uOsm may be both prognostic and predictive marker of therapy response in ADPKD patients. However, since water intake and the daily amount of osmoles excreted, which are known to determine uOsm, were not taken into account in this study, further studies are necessary to validate uOsm as a predictor of response to therapy in ADPKD.

Taken all together, both urinary osmolality and U/P urea are reliable surrogate biomarkers for maximal urine-concentrating capacity and, based on the analyzed findings, seem to have a clinical utility as predictors of a rapid ADPKD progression. Since implementation of these potential prognostic biomarkers in everyday clinical practice is limited by the scarce evidence, additional studies are required for validating uOsm and U/P urea as predictors of a future kidney functiondecline and tolvaptan treatment efficacy in ADPKD patients.

### Components of the renin-angiotensin system

Hypertension occurs in ADPKD patients even earlier than in patients with essential hypertension and development of an early-onset hypertension is an important predictor of renal outcome in ADPKD (11–14, 139). Intrarenal renin-angiotensin system (RAS) seems to have a central role in the development of hypertension in ADPKD since development and expansion of cysts activate intrarenal RAS before than renal fibrosis and renal function decline (140). Urinary angiotensinogen (uAGT) has been proposed as a surrogate biomarker of the intrarenal RAS activity (64, 141). UAGT levels correlate with renal function, proteinuria and hypertension in a wide range of CKD population (64, 65). Kocyigit et al. (66) demonstrated that uAGT is a biomarker of development and severity of hypertension in ADPKD patients. Since uAGT is too large molecule (52- to 64-kD) to be filtered through glomeruli, it hardly indicates ongoing processes in ADPKD kidneys, though it may reflect underlying pathophysiology. Indeed, AGT is highly expressed in cyst lining cells of ADPKD patients (140). This theory has been supported by a report of amelioration of cyst growth by suppression of AGT synthesis in PKD1 animal model (67).

Salih et al. (68) reported five- to six-fold higher levels of urinary AGT and renin excretions in ADPKD patients (*n* = 60) compared to sex, eGFR, blood pressure and RAAS inhibitor use matched non-ADPKD CKD patients (*n* = 57), while plasma levels of AGT and renin did not differ between the groups. Accordingly, higher urinary excretion levels of AGT and renin might be unique ADPKD features that are not simply a consequence of elevated plasma RAAS concentrations and renal function decline, but might be contributors to kidney damage. It must be noted that some of the included patients were on therapy with RAAS inhibitor and that there was a lack of age matching between ADPKD and non-ADPKD CKD patients. Lastly, even though significant, the strength of the associations detected in this study was modest, so additional studies in wider cohorts are warranted.

Park et al. (142) found that the urinary AGT to creatinine ratio (uAGT/Cr) correlates better with concurrent eGFR, htTKV, and hypertension than other biomarkers including urinary N-acetyl-β-D-glucosaminidase (NAG) and β-2-microglobulin (β2MG) in a cross-sectional observational research including 233 ADPKD patients. Urinary AGT/Cr levels in ADPKD patients started to increase in early disease stages (CKD stage I-II), suggesting that uAGT/Cr may reflect renal damage earlier than renal functional (eGFR) or radiological markers (htTKV) change. Plesinski et al. (69) reported a positive correlation between uAGT, uAGT/Cr and eGFR values in a cohort of 20 children with ADPKD, whereas similar relationships were not observed in the non-ADPKD group (18 children with a single cysts and one child with cystic dysplasia). However, there were no differences in serum or uAGT levels between those two groups, suggesting there is no increased expression of the RAAS system components in ADPKD pediatric population.

Kim et al. (70) confirmed significantly higher level of uAGT excretion in ADPKD patients (*n* = 293) than in non-ADPKD CKD patients (*n* = 1,495). Furthermore, the authors were the first to examine uAGT/Cr as a prognostic biomarker in ADPKD and found that, indeed, the uAGT/Cr ratio correlates positively with the risk of all-cause mortality and renal function decline in ADPKD patients, proposing its utility as a prognostic biomarker in ADPKD patients. However, this study did not include htTKV and PKD genotype as covariables. Moreover, since it takes a long time before ESRD or death occur in ADPKD (composite outcome in this study), it would be more convenient to analyze annual change in eGFR as a marker of renal function decline within a shorter (few years) follow-up duration.

The same group of authors evaluated uAGT/Cr as a prognostic biomarker for renal function decline in addition to imaging classification in a subsequent prospective study on 207 ADPKD patients with different CKD stages followed-up for more than one year (71). Patients were defined as slow (SP) or rapid (RP) progressors according to the Mayo classification and furtherly divided in high AGT or low AGT subgroup according to their uAGT/Cr values. Higher baseline uAGT/Cr emerged as an independent predictor of a faster eGFR decline, even after adjusting for gender, PKD genotype, baseline eGFR, blood pressure and albuminuria. Interestingly, uAGT/Cr showed an additional predictive value for renal outcome in slow progressors (Mayo classes 1A or 1B). Altogether, this study demonstrated an additional predictive value of uAGT/Cr for renal function decline in ADPKD patients beyond the Mayo classification. Of note, other components of the RAS system including plasma renin and aldosterone were not measured in this study and only baseline uAGT/Cr was measured with no repeated measurements. Therefore, future studies should elucidate whether repeated measurements of uAGT/Cr have a value in monitoring or predicting ADPKD progression.

Considering all the presented data, it is reasonable to conclude that uAGT is a valid early biomarker of ADPKD progression that reflects underlying pathophysiology. In addition to its individual prognostic value, combination of uAGT, imaging classification and/or genotype could be used to delicately define rapid ADPKD progressors. Nevertheless, additional studies validating utility of uAGT as a prognostic ADPKD biomarker are necessary before incorporating uAGT measurements in clinical settings.

### Fibroblast growth factor 23

Fibroblast growth factor 23 (FGF23), a bone-derived phosphaturic hormone, has been recognized as a strong risk factor for ESRD, cardiovascular morbidity and mortality in CKD patients (72). Pavik et al. (73) reported that patients with ADPKD have a fourfold increased serum FGF23 concentration even before renal function declines. Two later studies (74, 75) showed that patients with higher baseline serum FGF23 levels have a greater htTKV growth and eGFR decline rate, and an earlier onset of ESRD or death. An important difference between these two studies is that in the first study (74), which included 1,002 ADPKD patients followed-up for 5.4 years, FGF23 did not largely improve the prediction of a kidney function decline over htTKV, while, contrarily, in the second study (75), which monitored 192 ADPKD patients over 13 years, FGF23 emerged as a strong predictor of kidney function decline independently of htTKV or PKD genotype. The considerably longer follow-up period in the second study (13 years) may have given it more power to reveal temporal differences in disease outcomes.

To conclude, FGF23 is a potential prognostic biomarker for accelerated disease progression and early-onset undesirable clinical outcomes in ADPKD. Further studies are necessary to determine whether FGF23 improves prognostic value of commonly used prognostic models based on htTKV and PKD genotype as well as to elucidate whether the detected correlation between FGF23 and ADPKD outcome represents causal effect.

### Soluble urokinase plasminogen activator receptor

Hayek et al. (76) investigated association between a recently discovered marker of immune activation with a presumed role in the pathogenesis of kidney injury, soluble urokinase plasminogen activator receptor (suPAR), and ADPKD progression in 649 ADPKD patients followed up for at least 3 years. They found that high plasma suPAR levels significantly correlate with a kidney function decline and incident ESRD, independently of htTKV. Patients with the lowest suPAR levels (<2.18 ng/ml) had the slowest eGFR decline, whereas those with the highest suPAR values (>2.83 ng/ml) had the most rapid eGFR decline and a 3.38-fold higher risk of incident ESRD. Moreover, suPAR levels allowed distinguishing fast from slow disease progressors even in those considered to be at a low risk of progression to CKD by htTKV < 600 ml/m and MIC 1C. Finally, adding suPAR level to the MIC significantly improved predictive value for progress to CKD. These results indicate that suPAR could be useful for the early identification of ADPKD rapid progressors.

Since the study reviewed above is, to our knowledge, so far the only published research on suPAR in ADPKD patients, additional studies are necessary for validating suPAR as a prognostic biomarker in ADPKD.

### Somatostatin

Somatostatin (SST) inhibits cAMP production in kidney and, consequently, inhibits renal cyst cells proliferation (143, 144). Hence, it is not surprising that a few randomized clinical trials suggested that treating ADPKD patients with SST analogues may slow disease progression (35, 36). However, SST role in ADPKD pathophysiology and disease progression has not been supported by the results of Messchendrop et al. who were the first to investigate the association between plasma SST level and ADPKD severity and progression (145). In the latter study, baseline fasting plasma SST concentration did not correlate with urinary cAMP excretion, disease severity or disease progression evaluated by annual change in GFR and htTKV in a cohort of 127 ADPKD patients. These data suggest that plasma SST does not have a potential role as a prognostic biomarker in ADPKD.

### Serum bicarbonate

Blijdrop et al. (77) reported that in patients with ADPKD and eGFR 30–60 ml/min/1.73 m^2^ (*n* = 296, 2.5 years follow-up), serum bicarbonate is independently associated with kidney outcome (30% eGFR decline or kidney failure or a more rapid annual eGFR decline). Namely, each mmol/l serum bicarbonate decline increased the risk of kidney function deterioration by 21% and was associated with further eGFR decline. Serum bicarbonate was not associated with changes in htTKV. Intriguingly, serum bicarbonate correlated with kidney outcome independently of urinary ammonium excretion (a biomarker of urinary acidification capacity) as well as of variables included in the most commonly used ADPKD prognostic models, MIC and PROPKD score. The authors proposed lower serum bicarbonate (within the normal range) as a predictor of poor kidney outcomes in ADPKD. Further studies with a longer follow-up are warranted to validate these results as well as to examine whether there is a causal relation between low serum bicarbonate and an accelerated kidney function decline.

### Secreted frizzled-related protein 4

Secreted frizzled-related protein 4 (sFRP4), an antagonist of the Wnt signaling pathway, is highly expressed in kidneys, blood, cyst fluid and urine of ADPKD patients (146). Zschiedrich et al. (78) repeatedly measured serum sFRP4 levels in 429 ADPKD patients during 1.5 years follow-up in order to evaluate its prognostic utility. SFRP4 serum concentrations were elevated and correlated with the degree of renal failure. Moreover, increased baseline sFRP4 concentration predicted faster renal function decline. These findings indicate that serum sFRP4 levels might assist in recognizing ADPKD patients with a high risk of accelerated disease progression. Intriguingly, increased sFRP4 levels were noticed even in ADPKD patients with preserved renal function, suggesting sFRP4 levels might be helpful to identify rapid progressors in early stages of ADPKD disease. Considering the impressive results found in this isolated research, sFRP4 should definitely be the subject of future studies aiming at finding a prognostic biomarker for ADPKD.

### Uric acid

As with patients with CKD of other causes, hyperuricemia seems to be involved in the development and progression of ADPKD (147). Higher baseline serum uric acid in ADPKD patients has been associated with a higher incidence and earlier onset of hypertension and ESRD (79). In a study involving 680 ADPKD patients, Helal et al. (79) found that each mg/dl increase in uric acid is accompanied by a 5.8% increase in htTKV, even after adjusting for age, sex, and GFR. This finding was later validated in a cohort of 365 Korean ADPKD patients (80). Moreover, in the latter study, the annual eGFR decline decreased after the introduction of therapy aimed at lowering serum uric acid level (5.3 vs. 0.2 ml/min/ 1.73 m^2^ per year). These potentially important correlations must be verified in future large prospective clinical studies.

### Urine inflammatory, glomerular and tubular damage biomarkers

The urine concentration of molecules which are highly expressed in the settings of kidney injury or ischemia may be predictive of further renal function deterioration. These include indicators of tubular injury, such as the kidney injury molecule-1 (KIM-1), neutrophil gelatinase-associated lipocalin (NGAL) or β 2-microglobulin (β2MG) (148, 149). Likewise, molecules over-expressed under ischemic conditions, such as NGAL, vascular endothelial growth factor (VEGF) and monocyte chemoattractant protein-1 (MCP-1) might be biomarkers of cyst expansion and, therefore, disease progression (150). Most of these urinary molecules have been associated with ADPKD disease severity in a number of cross-sectional studies (81–84), yet longitudinal studies evaluating their prognostic value in ADPKD patients are limited by a small number and marked heterogeneity of used methods and accumulated results.

Messchendorp et al. conducted two consecutive studies evaluating urinary biomarkers as predictors of ADPKD progression (annual GFR or htTKV change) (85, 86). The first study (85) included 104 relatively young (40 ± 11 years) ADPKD patients with a preserved kidney function, while the second study (86) included 302 (152 in the longitudinal part) ADPKD patients (48.3 ± 7.43 years) in later disease stages. Urinary β2MG and MCP-1 excretion were both associated with annual change in GFR, even after adjusting for established risk factors (PKD mutation, baseline htTKV and eGFR). Urinary biomarker score created by summing β2MG and MCP-1 excretion tertiles was found to be superior to MIC and equal to PROPKD score in predicting accelerated renal deterioration. Segarra-Medrano et al. reported elevated urinary levels of markers of tubular injury (KIM-1 and β2MG) and renal ischemia (VEGF and MCP-1) and their significant correlation with htTKV and eGFR in 130 ADPKD patients (87). Multivariate model including urinary concentrations of β2MG, MCP-1 and VEGF improved the predictive value of htTKV for future eGFR decline. However, molecules examined in this study were not measured before observing a decline in the eGFR slope making it impossible to assess their utility as prognostic biomarkers in early ADPKD stages. Furthermore, the type of PKD mutation was not taken in consideration and spot urine samples instead of 24-h urine samples were used for all measurements. With these limitations on mind, further studies are necessary to confirm the observed correlations.

Meijer et al. investigated the association of urinary markers measured at baseline with eGFR decline in 46 ADPKD patients followed-up for 2.6 years (88). Baseline urinary excretion levels of albumin, IgG, KIM-1 and MCP-1 were inversely correlated with annual eGFR change, even after adjusting for age, gender, and baseline eGFR, whereas no such association was found for NGAL (88). More recently, Griffin et al. (89) also discerned baseline uKIM-1 as an independent predictor of a faster eGFR decline in 754 ADPKD patients. However, the latter association reached significance only in late ADPKD stages (eGFR < 60 ml/min/1.73 m^2^), with a trend towards significant correlation in early ADPKD. Urinary KIM-1 levels correlated with baseline HtTKV, but did not associate with annual change in HtTKV, suggesting that elevated uKIM-1 levels are not entirely caused by tubular injury due to cyst expansion. Of notice, htTKV measures were available only for patients in an early ADPKD stage. Based on the findings of these two studies (88, 89), urinary KIM1 seems worth further exploring as a predictor of ADPKD progression.

Although a number of reports support utility of NGAL as a prognostic biomarker in non-ADPKD CKD (151–154), results from longitudinal studies on ADPKD patients have not encouraged its prognostic value. Parikh et al. (90) did not find urinary NGAL to be associated with annual change in eGFR and TKV over 3 years in 107 ADPKD patients, indicating that NGAL is not useful in predicting renal outcome in ADPKD patients with preserved renal function. Contrarily, Viau et al. (91) reported increased uNGAL values in rapid (eGFR decline >4,5 ml/min/1,73 m^2^) compared to slow progressors in a cohort of 87 ADPKD patients. However, patients were in late stage of disease (eGFR 33 ± 20 ml/min/1.73 m^2^), making it possible that increased uNGAL levels were merely a consequence of a renal dysfunction. This hypothesis is further supported by the results of Boligano et al. (92) in which in 26 patients with late stage ADPKD urinary and serum NGAL levels were significantly higher in those with more advanced disease. Thus, NGAL might be a valuable biomarker of GFR decline during later stages of ADPKD.

Park et al. (155) reported no predictive value of urinary NAG for eGFR decline in a cohort of 270 ADPKD patients, although baseline NAG correlated significantly with both baseline eGFR and htTKV. Since the follow-up in the latter study was just 1 year long, further studies with longer follow-up duration should elucidate the value of urinary NAG for prediction of accelerated ADPKD progression.

In the aforementioned studies, urinary NGAL and NAG concentrations were measured in frozen-stored urine samples. It has been shown that frozen storage may lead to decreased measured concentrations of urinary biomarkers and increased variability resulting in reduced strength of associations (156). However, Schuh et al. (157) found only a minimal, clinically insignificant decrease (1%–3%) from baseline urinary NGAL, KIM1, and IL-18 levels after 3 freeze-thaw cycles and −80C storage for 5 years. Considering these reports, more research investigating urinary NGAL and NAG prognostic value in ADPKD is definitely needed.

Zimmerman et al. (93) reported an increased total number of intrarenal CD4 and CD8 T cells and a correlation between urinary CD4+ T cell count and annual eGFR decline over 5 years in a small cohort of ADPKD patients (*n* = 30), suggesting this novel marker as a candidate prognostic biomarker in ADPKD and supporting the proposed role of T cells in ADPKD pathogenesis. Li et al. (94) reported increased number of CD206 + macrophages in kidneys and urine from 30 ADPKD patients. The number of CD206 + macrophages correlated with the annual eGFR decline, indicating its utility for assessing disease activity and predicting renal function decline in ADPKD patients. Zhou et al. (95) demonstrated that an established marker of alternatively activated macrophages, CD14, is eminently up-regulated in ADPKD kidneys. Surprisingly, in ADPKD, increased CD14 levels originate from the cystic and non-cystic renal tubular epithelial cells instead of infiltrating macrophages. Furthermore, cyst fluid and immunoprecipitated urine from a small number of ADPKD patients (*n* = 16) showed increased expression of soluble forms of CD14. In this small group of ADPKD patients, baseline urinary CD14 levels (but not GFR) significantly correlated with a two-year rate of TKV change, indicating that urinary CD14 level could serve as a predictor of ADPKD outcome. Despite the strong correlations detected by flow cytometry analyses in these studies (93–95), the reported results are limited by small cohorts of ADPKD patients and, as a consequence, lack of the multiple covariate effects evaluation. Therefore, additional flow cytometry analyses in larger, well-characterized cohorts of ADPKD patients are required for validation of the reported findings and drawing conclusions.

Jones et al. (96) evaluated asymptomatic pyuria (AP) as a prognostic biomarker in a retrospective cohort study including 687 ADPKD patients: 342 with AP (AP group) and 340 with no pyuria (NP group). Patients with AP reached kidney failure at younger age compared to those with NP. Furthermore, occurrence of AP was associated with faster eGFR decline but not with the change in annual Ht-TKV rate of growth, meaning that renal function deteriorated after incident AP without considerable change in the rate of Ht-TKV growth. Authors concluded that occurrence of AP predicts accelerated ADPKD progression irrespective of the cystic burden and cystic growth. Further prospective studies are necessary to validate AP as a prognostic biomarker to predict faster disease progression in ADPKD patient.

Piazzon et al. (97) evaluated the diagnostic and prognostic value of Fetuin-A, a multifunctional negative acute phase protein, as a novel urinary biomarker of ADPKD. Fetuin A was significantly higher expressed in urine from ADPKD patients (*n* = 66) than in healthy controls (*n* = 17) or patients with other renal diseases (*n* = 50). Moreover, in ADPKD patients, urinary Fetuin-A level significantly correlated with the CKD stage. Most importantly, in a cohort of early-stage ADPKD patients, urinary Fetuin-A concentrations significantly increased during 2 years of follow-up without a concomitant increase in eGFR, suggesting that the urinary level of Fetuin-A might be a more sensitive biomarker for detecting disease progression than eGFR. Limitations of this study include the lack of PKD genotyping and TKV assessment. Further studies should investigate the pathogenic mechanisms of increased Fetuin-A expression in ADPKD.

Kim et al. (98) revealed glycoprotein osteopontin (OPN), a soluble cytokine that can be identified both in urine and plasma, as a potential urinary biomarker for predicting ADPKD progression. Urinary OPN levels were found to be lower in rapid ADPKD progressors (*n* = 11) compared to slow progressors (*n* = 11). Since all included patients had eGFR > 60 ml/min/1.73 m^2^, the authors suggested OPN is as an early biomarker of a rapid ADPKD progression. Interestingly, opposite to its decreased expression in ADPKD population, OPN expression in non-ADPKD CKD is increased (158). These intriguing findings make osteopontin worth further exploring in the context of ADPKD pathophysiology and progression.

Advantages of urinary biomarkers as predictors of accelerated ADPKD progression compared to currently used PKD genotyping and repeated measurements of TKV are easier analysis, wider availability and lower cost. Furthermore, the proposed urinary biomarkers may also be useful to detect any additional kidney damage (due to lifestyle factors or comorbidities) on top of that caused by ADPKD, unlike htTKV growth or PKD mutation which are specific for ADPKD. This might lead to a more individualized approach to ADPKD patients. Additional advantage of urinary biomarkers compared to TKV and PKD mutation is a rapid response to treatment allowing better therapeutic guidance. However, studies investigating whether baseline or treatment-induced short-term changes in urinary biomarkers correlate with long-term outcome are lacking.

To summarize, among a large number of urinary molecules investigated in the studies reviewed above, urinary β2MG, MCP-1, KIM1 and VEGF have been discerned in most of the studies as the ones with the best predictive value for a rapid ADPKD progression (85–89). Still, each of those biomarkers separately showed relatively low predictive value for future ADPKD progression. This indicates that to accurately predict prognosis in ADPKD, different biomarkers should be used together as a urinary biomarker score or in combination with genotype- or kidney volume- based models, to achieve adequate risk prediction. Furthermore, flow cytometry analyses of the urine specimens from ADPKD patients proposed urinary CD4+ T cell (93), CD206 + resident macrophages (94) and CD14 +mononuclear cells (95) counts as potential predictors of ADPKD progression. Finally, asymptomatic pyuria, Fetuin A and osteopontin were shown to have a high predictive value for a fast ADPKD progression in one isolated study each (96–98). Therefore, all of these biomarkers are seeking for validation in additional studies. Moreover, due to heterogeneity of measurement methods and rapid progression definitions used in the reviewed articles, it is reasonable to conclude that further, more uniform studies, should elucidate the most reliable marker among the proposed candidates for predicting disease progression in ADPKD.

### Telomeric epigenome

Kocygit et al. are the first to explore the link between telomeric epigenome and ADPKD progression (99). ADPKD patients (*n* = 78) were found to have a significant shortening of telomere length (TL) and higher levels of RNA containing telomere repeat (TERRA) levels compared to healthy controls (*n* = 20). Moreover, 16 genetically unrelated patients with rapidly progressive ADPKD demonstrated higher levels of TERRA with shorter telomeres compared to slow progressors. Interestingly, genetically related individuals carrying the same PKD mutation differed in TL and TERRA levels, independently of gender and age. The reported intrafamilial variations emphasize a potential role of these epigenetic biomarkers in predicting the progression of ADPKD. Due to the intriguing correlations observed in this isolated study of TL and TERRA in a small cohort of ADPKD patients, further studies could be of a great significance for individualized approach and better management of ADPKD patients.

### Metabolomic biomarkers

Since conventional markers have numerous limitations, metabolic profiling holds a promise as biomarker for risk categorization of ADPKD patients. A small cross-sectional study by Gronwald et al. (159) demonstrated that urinary metabolic profiling may serve for distinguishing ADPKD patients with preserved renal function from those with impaired renal function and healthy volunteers.

Furthermore, Rowe et al. (160) identified elevated levels of key glycolytic enzymes (lactate dehydrogenase A [LDHA], pyruvate dehydrogenase kinase 1 [PDK1], and the pyruvate kinase M2 [PKM2] isoform) in ADPKD cystic epithelial cells as compared with normal kidney epithelial cells, indicating shift from oxidative to glycolytic fluxes in ADPKD. Hallows et al. (100) found a positive association between urinary excretion of PKM2 and LDHA and ADPKD severity (assessed by htTKV and eGFR) in 95 ADPKD patients (161), thus providing evidence of up-regulated glycolytic flux as a feature of ADPKD severity. In a subsequent longitudinal study (101) Hallows et al. found that urinary lactate/pyruvate ratio, an enhanced glycolytic and reduced oxidative metabolic marker, is significantly inversely correlated with the risk of disease progression (eGFR decline), suggesting its utility as prognostic ADPKD biomarker. These findings provide additional evidence for the assumed dysregulated metabolism with increased glycolytic flux as a correlate of ADPKD severity. The correlations found in these two studies are limited by the small sample size as well as a lack of standardization of urine collection times and fasting status.

Dekker et al. (102) singled out four urinary metabolites (myo-inositol, 3-hydroxyisovalerate, ADMA and creatinine) robustly correlated with disease severity (assessed by actual eGFR) by targeted urinary metabolic profiling of 501 ADPKD patients. Furthermore, the authors discerned urinary alanine/citrate ratio as the best predictor of future disease progression (annual rate of eGFR decline). Although this association was weak (R2 50.15), the urinary alanine/citrate ratio improved the prognostic performance of conventional risk markers including htTKV. These findings are limited by a short follow-up time of 2.5 years what might have reduced the value of the eGFR slope in predicting long-term disease progression and ESRD. Additionally, the validation cohort in this study consisted only of patients with later-stage CKD. Hence, additional studies in larger cohorts and with a longer follow-up are necessary to verify the value of the suggested ratio as a biomarker for predicting rapid disease progression.

In a subsequent study, Dekker et al. (103) validated the prognostic performance of the previously proposed urinary metabolites measured at a single time point to distinguish those with accelerated disease progression in a cohort of 324 ADPKD patients. Moreover, in a subset of 112 ADPKD patients with a 3-year follow-up, fast progressors (annual eGFR change > −3.0 ml/min/1.73 m^2^/year) demonstrated an increase in the urinary myoinositol/citrate ratio whereas no significant change in the myoinositol/citrate ratio was found in slow progressors. The prognostic value of the urinary myoinositol/citrate ratio in predicting rapid disease progression was at least comparable to that of the conventional risk marker htTKV.

Baliga et al. (104) performed targeted metabolomics to characterize and compare plasma fingerprints (metabolomic profile) between 58 pediatric ADPKD patients with preserved kidney function and 98 age-matched healthy controls. Furthermore, the authors designated methyladenosine, ornithine and kynurenate as the three metabolites significantly associated with both baseline HtTKV and the change in HtTKV during 3 year follow-up. Since the reported associations are limited by the absence of a disease control group that received no treatment (all participants received ACE inhibitors) and a lack of PKD genotyping in participants, the proposed three metabolites should be verified as ADPKD prognostic biomarkers in additional prospective clinical studies. In a subsequent study (105), the same author group verified the suggested role of kynurenins in predicting ADPKD progression. However, in this study kynurenine and kynurenic acid, even though positively associated with disease severity (baseline htTKV and eGFR), did not correlate with disease progression (annual change in htTKV or eGFR).

Kang et al. (106) performed targeted metabolomics and identified nine progression-related biomarkers in serum samples from 124 ADPKD patients: hexadecenoylcarnitine, pimelylcarnitine, creatinine, acyl-alkyl-phosphatidylcholin and five different diacyl-phosphatidylcholines. Prognostic model incorporating selected metabolomic biomarkers and clinical parameters demonstrated higher predictive value for PKD progression than clinical parameters alone. Metabolites identified in this study could help in distinguishing PKD patients with a higher risk of rapid disease progression and in a need of aggressive treatment, though validation in further studies is required.

In summary, each of the reviewed metabolomic studies among ADPKD patients proposed different metabolites for predicting rapid ADPKD progression, and those are: urinary lactate/pyruvate ratio (101), urinary alanine/citrate ratio (102), urinary myoinositol/citrate ratio (103), urinary methyladenosine, ornithine and kynurenate levels (104, 105) and a prognostic model incorporating hexadecenoylcarnitine, pimelylcarnitine, creatinine, acyl-alkyl-phosphatidylcholin and five different diacyl-phosphatidylcholines (106). Since metabolic profiling seems to be a promising strategy for predicting disease progression in ADPKD patients by reflecting the underlying dysregulated metabolism with increased glycolytic flux, all of the suggested metabolites should be verified as ADPKD prognostic biomarkers in additional prospective clinical studies.

### Proteomic biomarkers

Proteomics, or the study of small peptides which compose a sample, has become a method widely applied in different fields of research. It comes as no surprise therefore that urinary proteomics have a promising potential for discovering novel biomarkers in ADPKD. Kistler et al. (107) were the first to perform capillary electrophoresis coupled to mass spectrometry (CE-MS) analysis to identify peptide markers for ADPKD or so-called urinary proteomic “footprint” of ADPKD. A panel constructed with the identified 142 ADPKD-associated peptides (ADPKD_142 urinary biomarker model) was highly specific for ADPKD when applied in two independent validation cohorts. The majority of the selected urinary biomarkers were fragments of collagen, supporting hypothesis of extracellular matrix destruction by cyst development and expansion. The ADPKD_142 score correlated significantly with baseline htTKV, annual htTKV growth and eGFR. Since those correlations were weak, authors proposed and validated another proteomic severity score (99 peptides) that strongly correlates with htTKV in ADPKD patients (107). In a longitudinal study involving 221 ADPKD patients (108), urinary proteome analysis resulted in an invention of another panel consisting of 20 selected peptides. This panel allowed prediction of a risk of progression to ESRD within 13 years in ADPKD patients older than 24 years and a 30 ml/min/1.73 m^2^ GFR decline over 8 years in patients younger than 24 years. The performance of this biomarker panel was comparable to that of htTKV and the combination of the biomarker panel with htTKV demonstrated the highest predictive value for ADPKD progression to ESRD.

Recent progress in “omic” studies has facilitated the discovery of extracellular vesicles (EVs) as novel biomarkers allowing the detection of pathologic processes even earlier than conventional biomarkers (162). Indeed, multiple gene products associated with ADPKD have been identified in urine EVs (163, 164). Hogan et al. (109) were first to report that urinary exosome proteomic profiling evidently distinguishes between healthy subjects and ADPKD patients. ADPKD were found to have lower levels of polycystin-1 (PC1) and polycystin-2 and higher levels of transmembrane protein 2 (TMEM2) compared to healthy controls. The PC1/TMEM2 ratio in ADPKD patients was inversely correlated with htTKV, suggesting that patients with unfavorable outcomes may have low PC1/TMEM2 ratios from the disease beginning. Salih et al. (110) reported that younger ADPKD patients with preserved kidney function have increased levels of complement C3 and C9 in urinary EVs, whereas patients with advanced ADPKD express higher levels of villin-1, periplakin, and envoplakin. Raby et al. (111) analyzed urinary exosomal proteome in 30 patients with various stages of ADPKD followed-up for 10 years to investigate its potential to discern patients with a future rapid disease progression (eGFR decline > 10 ml/min). The most differentially expressed proteins between the two groups were microtubule-associated serine/threonine kinase (MAST)-4 (24x), cytokinesis-associated kinesin-like (KIF) 20B (13x), and dynein heavy chain (8x). In addition, patients with a good response to a 4 years long treatment with tolvaptan showed increased expression of Wnt/*β*-catenin and PDGF-signaling proteins in their urinary EVs at baseline, whereas those with a poor response to therapy had highly expressed angiogenesis pathway proteins.

Even though proteomic expression profiling might be superior to any other biochemical marker in predicting disease progression and response to therapy in ADPKD patients, incorporating urinary proteomics in everyday clinical practice has some challenges, including variations in the urine proteome based on multiple factors, such as age, sex, diet, and physiological state (165). Furthermore, isolation and purification of EVs still represents a challenge because of the interference of similar size molecules, such as high-density lipoproteins, chylomicrons, and microvesicles (166). Hence, optimizing methods for isolating/purifying EVs is mandatory before introducing them as biomarkers in clinical practice. Finally, many centers still lack laboratory facilities where such analysis could be performed.

Taken all together, the proteomic studies on ADPKD patients are only at the beginning. Despite the marked heterogeneity between the design and the results of the studies reviewed above, the observed correlations strongly advocate proteomic profiling as a promising tool for discerning patients with a future rapid ADPKD progression and for identifying those among rapid progressors who will doubtlessly benefit from treatment with tolvaptan. Further proteomic studies in ADPKD patients are indispensable for revealing reliable ADPKD prognostic biomarkers reflecting the underlying pathophysiological pathways. In addition, development of a human protein atlas, especially urinary exosomal protein expression atlas (167), will assist therapeutic decision-making in ADPKD and contribute to a more personalized management of ADPKD patients.

### MicroRNAs

As with proteomics, microRNAs, small highly conserved non-coding RNA molecules with role in gene expression regulation, have also become an important subject of numerous basic and translational research, and ADPKD is no exception. Kocygit et al. (112) reported that ADPKD patients have higher serum levels of miR-3907-3p, miR-92a-3p, miR-25-3p and miR-21-5p, and lower levels of miR-1587-5p and miR-3911-5p compared with healthy controls. Moreover, these levels correlate with the degree of renal function decline. On top of all that, increased serum levels of miR-3907-3p are associated with a more rapid ADPKD progression. Ben-Dov et al. (113) revealed decreased levels of mir-1(4) and mir-133b(2) in ADPKD patients compared to non-ADPKD CKD patients, suggesting their involvement in ADPKD pathogenesis. Intriguingly, several microRNAs were detectable only in ADPKD patients. Magayr et al. (114) reported that 5 miRNAs (miR-192-5p, miR-194-5p, miR-30a-5p, miR-30d-5p and miR-30e-5p) are significantly down-regulated in ADPKD urine EVs as well as in cystic kidney tissue. All five miRNAs showed significant correlation with baseline eGFR and mean kidney length (MKL). MiR-30e-5p showed the highest predictive value for a rapid ADPKD progression (annual eGFR decline > 3 ml/min/year). Combination of all five microRNAs showed higher predictive value for a faster disease progression than MKL. However, the combination of both miRNAs and MKL correlated even better with the rate of future disease progression.

MicroRNAs proposed as diagnostic and prognostic ADPKD biomarkers in these studies (112–114) require validation in different cohorts of ADPKD patients in future studies. On top of that, optimization of miRNA profiling of human specimens is needed for implementation of miRNAs as prognostic biomarkers in clinical practice.

## Conclusion

Despite tremendous effort to elucidate all aspects of ADPKD through extensive basic and translational research, there is still an unmet clinical requirement for biomarker(s) which could predict the rate of disease progression, either alone or in combination with the conventional prognostic markers in ADPKD patients. The recent entrance in the era of targeted therapies in ADPKD has emphasized the urgent need to identify reliable and widely accessible biomarkers for predicting ADPKD progress. Identification of such biomarker would allow optimal patient selection for an early introduction of therapy or inclusion in clinical trials.

The studies incorporated in this review demonstrate the multifaceted character of the pursuit of identifying the most reliable prognostic marker for ADPKD. We focused on articles investigating markers that can simply be measured in patients' blood or urine samples since these are the most widely available tools. According to studies reviewed in the presented manuscript, the most promising ADPKD prognostic biomarkers are molecules involved in the disease pathogenesis: markers of tubular injury, inflammation, metabolism, the renin-angiotensin or the vasopressin system. However, multiple ADPKD pathogenic pathways and involved molecules are yet to be exposed. Although none of the proposed prognostic biomarkers has shown a prominent additional prognostic value beyond kidney volume- or genotype-based prognostic models, several studies provided evidence that incorporating certain serum or urine biomarker and Mayo classification or PROPKD score might be the most accurate risk prediction model for a rapid disease progression in patients with ADPKD. Among various molecules under investigation, the most promising ones for predicting ADPKD progression are copeptin, apelin, angiotensinogen, FGF23, suPAR, serum bicarbonate, sFRP4, KIM1, MCP1, β2MG, CD14, fetuin A, osteopontin, along with asymptomatic pyuria, urine osmolality and urine to plasma urea ratio. Despite numerous studies comparing these biochemical markers, to identify the finest for predicting ADPKD progression, many studies are still lacking. Moreover, in the “omics” era, multiple additional candidates are emerging as highly specific ADPKD prognostic biomarkers hopefully leading to individualized approach and personalized risk assessment and therapeutic decision-making in ADPKD patients.

Even though ADPKD is increasingly diagnosed in pediatric age, studies investigating biomarkers of ADPKD progression in children are surprisingly scarce. Among the 73 studies thoroughly analyzed in this review, only 4 studies (51, 69, 104, 105) were conducted on pediatric ADPKD population with the results limited by a small sample size. Obtaining urinary biomarker concentration is a non-invasive procedure, making such investigations appropriate for the pediatric population. Among the urinary biomarkers, those that seem worth further exploring in terms of ADPKD progression in children are urinary copeptin, urine to plasma urea ratio, urine osmolality, uAGT, uKIM1, urinary β2MG and MCP-1. Additionally, urinary proteomic and metabolomic analyses in children might enlighten the underlying pathophysiologic pathways leading to early-onset ESRD. With the more and more frequently met question of an early introduction of renal-protective agents in children with ADPKD, the need for studies aiming at finding a reliable early biomarker for therapy guidance has been emphasized.

In conclusion, the available data are not sufficient to draw the final conclusions. Although the proposed molecules show promising results that warrant further investigation, scarce evidence limit their potential to displace traditional biomarkers in the near future. Additional studies with large cohorts and long follow-up are necessary to determine the superiority of proposed biomarkers to traditionally used ADPKD prognostic models. Hopefully, the emerging evidence from studies with large ADPKD cohorts will allow for some of the herein mentioned molecules to be used in everyday clinical practice for predicting ADPKD progression and therefore improving disease management.
